# Ultrasonic scalpel causes greater depth of soft tissue necrosis compared to monopolar electrocautery at standard power level settings in a pig model

**DOI:** 10.1186/1471-2482-12-3

**Published:** 2012-02-23

**Authors:** Kia Homayounfar, Johanna Meis, Klaus Jung, Bernd Klosterhalfen, Thilo Sprenger, Lena-Christin Conradi, Claus Langer, Heinz Becker

**Affiliations:** 1 Department of General and Visceral Surgery, University Medical Center Goettingen, Georg-August-University, Robert-Koch-Strasse 40, 37075 Goettingen, Germany; 2 Department of Medical Statistics, University Medical Center Goettingen, Georg- August-University, Humboldtallee 32, 37075, Goettingen, Germany; 3 Institute of Pathology, Roonstr. 30, 52351 Dueren, Germany; 4 Department of General, Visceral, Thoracic and Minimal-invasive Surgery, Evangelic Hospital Goettingen-Weende, An der Lutter 24, 37075 Goettingen, Germany

## Abstract

**Background:**

Ultrasonic scalpel (UC) and monopolar electrocautery (ME) are common tools for soft tissue dissection. However, morphological data on the related tissue alteration are discordant. We developed an automatic device for standardized sample excision and compared quality and depth of morphological changes caused by UC and ME in a pig model.

**Methods:**

100 tissue samples (5 × 3 cm) of the abdominal wall were excised in 16 pigs. Excisions were randomly performed manually or by using the self-constructed automatic device at standard power levels (60 W cutting in ME, level 5 in UC) for abdominal surgery. Quality of tissue alteration and depth of coagulation necrosis were examined histopathologically. Device (UC vs. ME) and mode (manually vs. automatic) effects were studied by two-way analysis of variance at a significance level of 5%.

**Results:**

At the investigated power level settings UC and ME induced qualitatively similar coagulation necroses. Mean depth of necrosis was 450.4 ± 457.8 μm for manual UC and 553.5 ± 326.9 μm for automatic UC versus 149.0 ± 74.3 μm for manual ME and 257.6 ± 119.4 μm for automatic ME. Coagulation necrosis was significantly deeper (*p *< 0.01) when UC was used compared to ME. The mode of excision (manual versus automatic) did not influence the depth of necrosis (*p *= 0.85). There was no significant interaction between dissection tool and mode of excision (*p *= 0.93).

**Conclusions:**

Thermal injury caused by UC and ME results in qualitatively similar coagulation necrosis. The depth of necrosis is significantly greater in UC compared to ME at investigated standard power levels.

## Background

Soft tissue dissection is a major issue in all fields of surgery as it incorporates the risk of wound healing disorder, hematoma or seroma. These adverse events potentially cause additional interventions up to reoperation resulting not only in patients discomfort and prolonged hospital stay but also in persisting morbidity and higher health care costs [1]. The search for a dissection tool safer than standard monopolar electrocautery (ME) with its well known limitations in particular burns and carbonization, has led to the development of high-frequency ultrasonic dissection tools (UC). These instruments transform electrical power into ultrasonic waves of 55.5 kHz. The subsequently released thermal energy breaks up protein molecules leading to hemostasis and tissue dissection by cavitation and coaptation with good controllability of penetration depth [2]. Numerous studies have evaluated the safety and feasibility of UC [3-7] and it has already been introduced into clinical routine in various subspecialties of surgery especially for laparoscopic procedures. However, there is still controversy about its potential advantages. Given the anyway limited vision in laparoscopic surgery the use of UC is favourable because of less smoke and reduced risk of thermal injury to adjacent structures as known from ME due to direct burns or capacitive coupling [5,8]. The higher costs for UC devices could be compensated by saving operating time as demonstrated for laparoscopic cholecystectomy, especially when UC is solely used [9].

However, besides its potential advantages data on the extent of tissue alteration and its potential adverse effects are inconsistent. The discussion on UC has been raised again since recent studies identified a higher rate of sexual disorders after laparoscopic rectal resection compared to open procedures [10] where to UC may contribute. Given that tissue alteration processes are different between UC and ME we hypothesized that these dissection tools also differ in quality and extent of tissue alteration. As UC has been shown to depend not only on power level setting but individual activation time and pressure [1,11], an experimental setup with a standardized tissue dissection technique without manual handling bias is needed to investigate the impact of UC on soft tissue morphology in comparison to standard ME. Therefore, we stepped back into an experimental pig model aiming to histopathologically evaluate the quality and extent of morphologic changes caused by UC and ME for soft tissue dissection using 2 types of dissection (manual and automated).

## Methods

Animal experiments were performed in 16 male 3-6 months-old pigs with a mean weight of 44.0 ± 4.7 kg. All animal care and experimental procedures were in accordance with German national legislation on animal protection and approval was given by the Ministry of Agriculture, the Environment and Rural Areas of Land Schleswig-Holstein, Germany (V 312-72241.123-34). The animals were anesthetized using the following sedation, relaxation, and narcosis regimen: ketamine 10% with a dose of 0.25 mL/kg, xylazine 2% in a dose of 0.15 mL/kg, atropine sulfate 1% in a dose of 0.06 mL/kg. After endotracheal intubation anesthesia was continued with constant isoflurane (1.5-2 vol%) inhalation and oxygen (50 vol%) with a fresh gas flow rate of 1 L/min.

**Figure 1:**
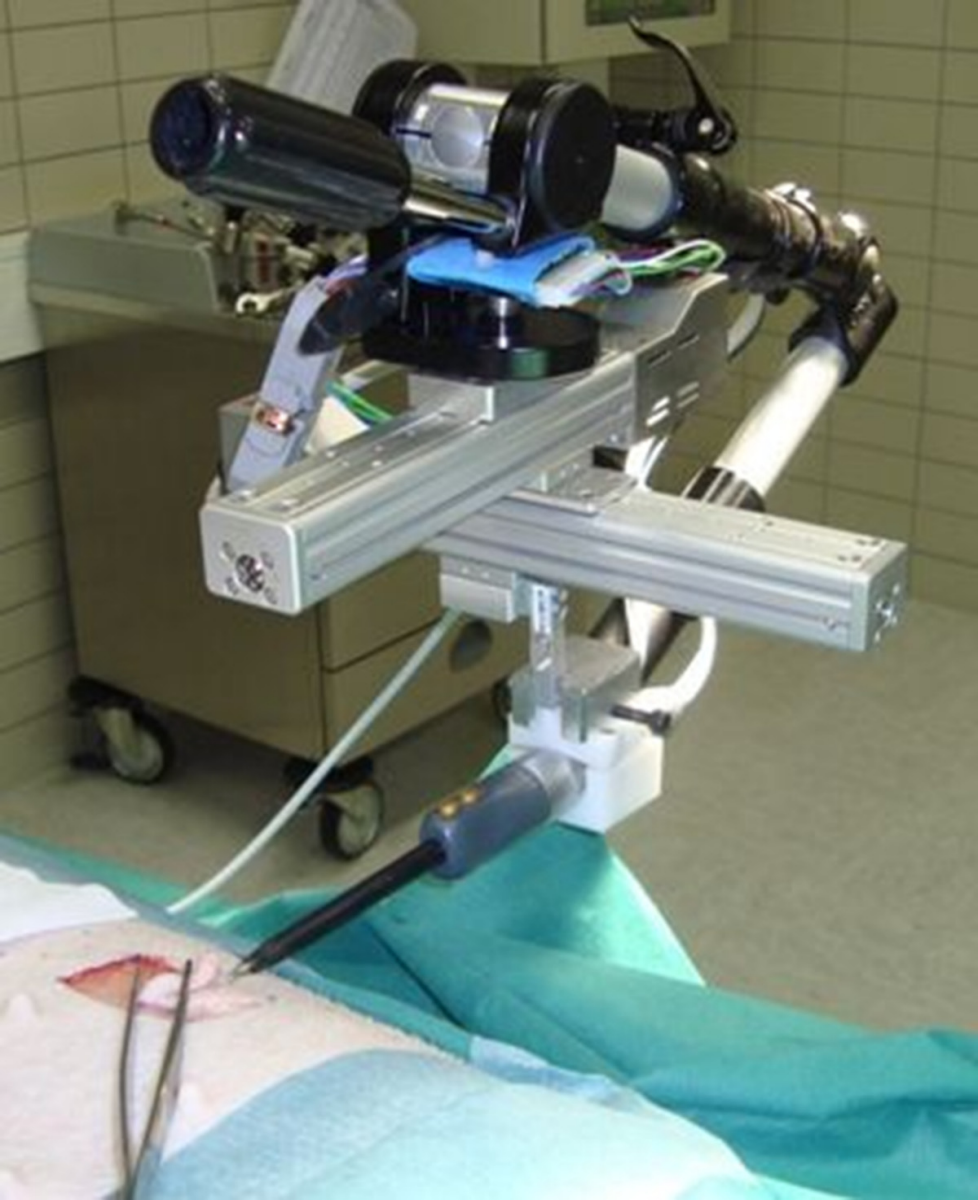
**Automatic device for soft tissue dissection**. Self-constructed apparatus fixed at the operating table and loaded with ultrasonic scalpel. The instrument can be moved engine-driven into two directions (aluminium tracks).

A software-controlled device was constructed for standardized automatic tissue dissection (Figure 1). After fixation at the operating table and insertion of the selected dissection tool, the device allowed identical excisions with fixed tissue contact times. Therefore, the dissector blade cut 5 cm in horizontal direction starting at an edge of the defined tissue sample and then moved forward for 1 cm redoing the same movements backwards until a 5 × 3 cm tissue sample was excised. In this study, we used the *Ultracision Harmonic Scalpel HSA07 (Ethicon Endo-Surgery, Inc, Nordestedt, Germany*.) on power level 5 for UC and the *Erbotom ICC 350 (ERBE Elektromedizin GmbH Tübingen, Germany*) on 60 W (cutting) for ME. These power level settings were chosen as they represent a widely used standard in abdominal surgery. While manual excision was performed in all 16, the automatic device was used only in 9 animals after validation experiments (data not shown).

**Figure 2:**
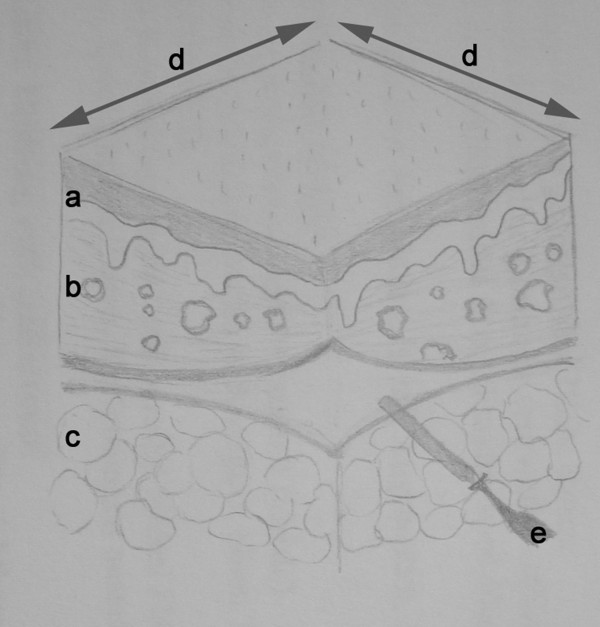
**Schematic illustration of tissue sample and excision planes**. **a**) epidermal layer, **b**) corium, **c**) subcutaneous fatty tissue, **d**) vertical excision lines performed by steel scalpel, **e**) horizontal excision line performed by either ultrasonic scalpel (UC) or monopolar electrocautery (ME).

Using a template, 8 excisions were sketched on each pig's abdominal wall. Then a double step randomization process defined the mode of excision (automatic versus manual) and the tissue dissection tool (UC versus ME) for each sample. Figure 2 illustrates the subsequent excision process. Vertical incisions were performed with a steel scalpel. Afterwards the tissue sample was excised in horizontal direction either by UC or ME, which exactly had to be done at the cutaneous-subcutaneous junction. Tissue samples were then fixed, dehydrated and paraffin embedded (*Leica TP1050 Tissue Processor, Leica EG 1140 Embedding Center, Leica Microsystems, Germany*). 3 micrometer cross sections of each sample were produced (*Sliding microtome, Leica Microsystems, Germany*) for Hematoxilin and eosin (HE) and Elastica van Gieson (EvG) staining. Light microscopy was performed by an experienced histopathologist (BK). 7 measure points were used to determine the median depth of necrosis in each sample. Preparation as well as histopathological and morphometric examination of all specimens was performed at BMP Labor für Medizinische Materialprüfung GmbH, Aachen, Germany using standard operating procedures and an accredited quality management system.

The influence of the variables dissection mode (automatic versus manual) and dissection tool (ME versus UC) onto the depth of necrosis was evaluated using a two-way analysis of variance for repeated measures, including the interaction effect of the two factors as well. Effects were found to be significant if *p*-values were less than 0.05. All analyses were performed using the free software R (version 2.8, http://www.r-project.org).

## Results

### Histological findings

**Figure 3:**
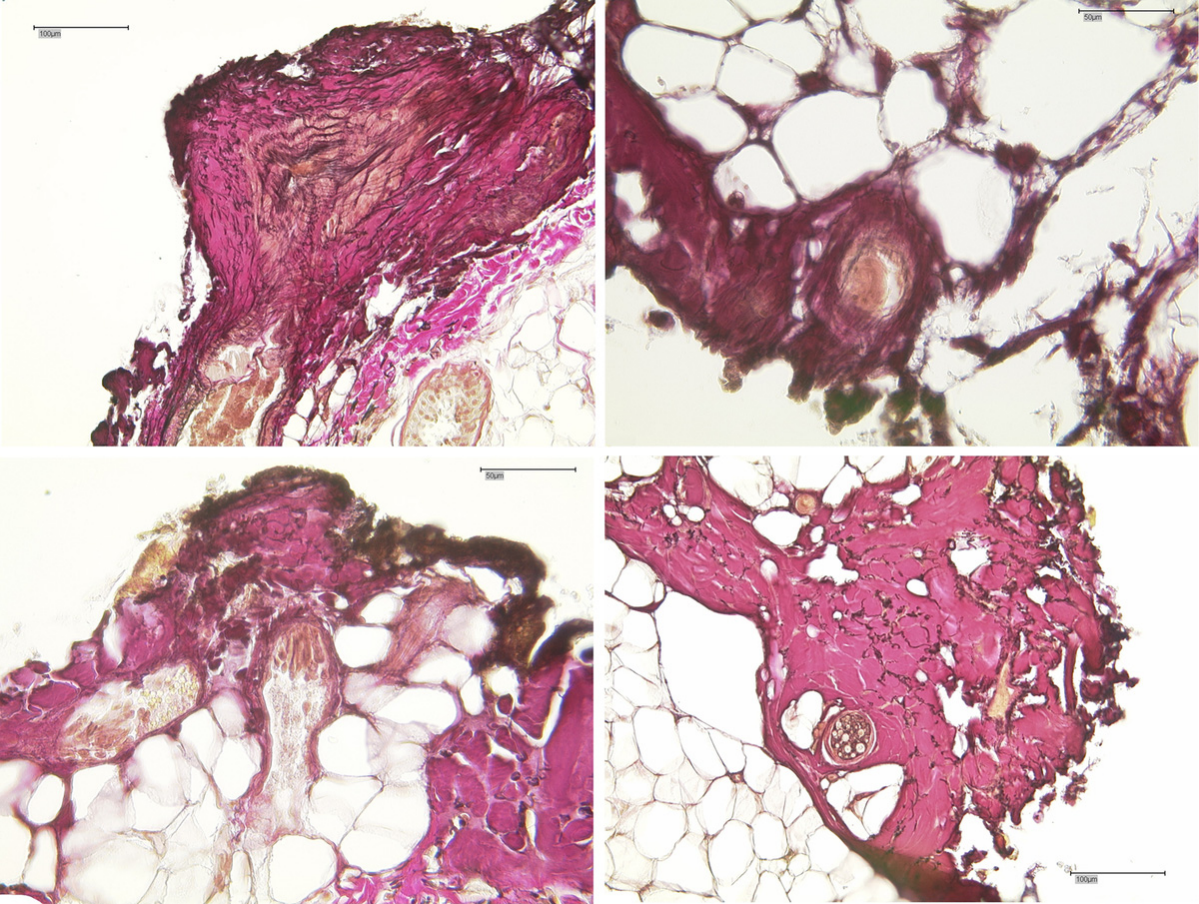
**Elastic-Van Gieson (EvG) staining**. Representative EvG staining results with deep red necrosis (**a**), more superficial necrosis with closure of a capillary (**b**) and deep necrosis with closure of a larger vessel (**c**) all caused by UC and necrosis with closure of small vessels caused by ME (**d**) (magnification a and d ×200; c and d × 400).

Conventional light microscopy using HE and EvG staining verified skin and subcutaneous tissue with a regularly structured epidermis of keratinized stratified squamous epithelium and stratum corneum with typical integumentary appendages in all 100 samples. The subcutaneous fat tissue consisted of univacuolary lipocyts as well as vessel-bearing connective tissue strings. Tissue samples of both, UC and ME showed qualitatively similar coagulation necroses at the resection plane which were pronounced in the fibrovascular connective tissue structures of the corium in relation to the subcutaneous fatty tissue. In addition, the consistence of the fatty tissue caused inaccuracy of depth extension determination. Therefore, further morphometric measurements of necrosis depth were restricted to that fibrovascular connective tissue layer. Figure 3 shows representative EvG staining results with deep red necrosis (a), more superficial necrosis with closure of a capillary (b) and deep necrosis with closure of a larger vessel (c) all caused by UC and necrosis with closure of small vessels caused by ME (d).

### Morphometric measurements

**Figure 4:**
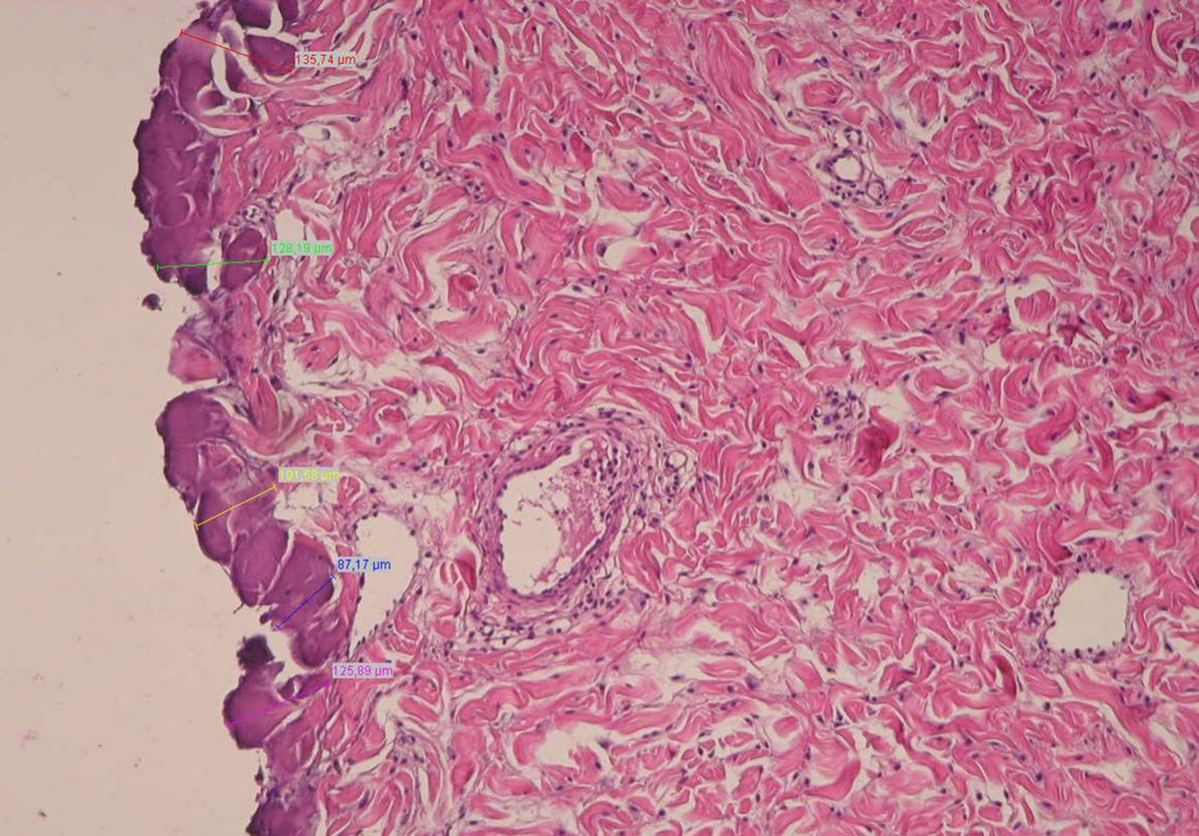
**Morphometric measurement**. Representative tissue section (HE staining) at ×100 magnification displaying the morphometric measurement with 7 different measuring points.

**Figure 5:**
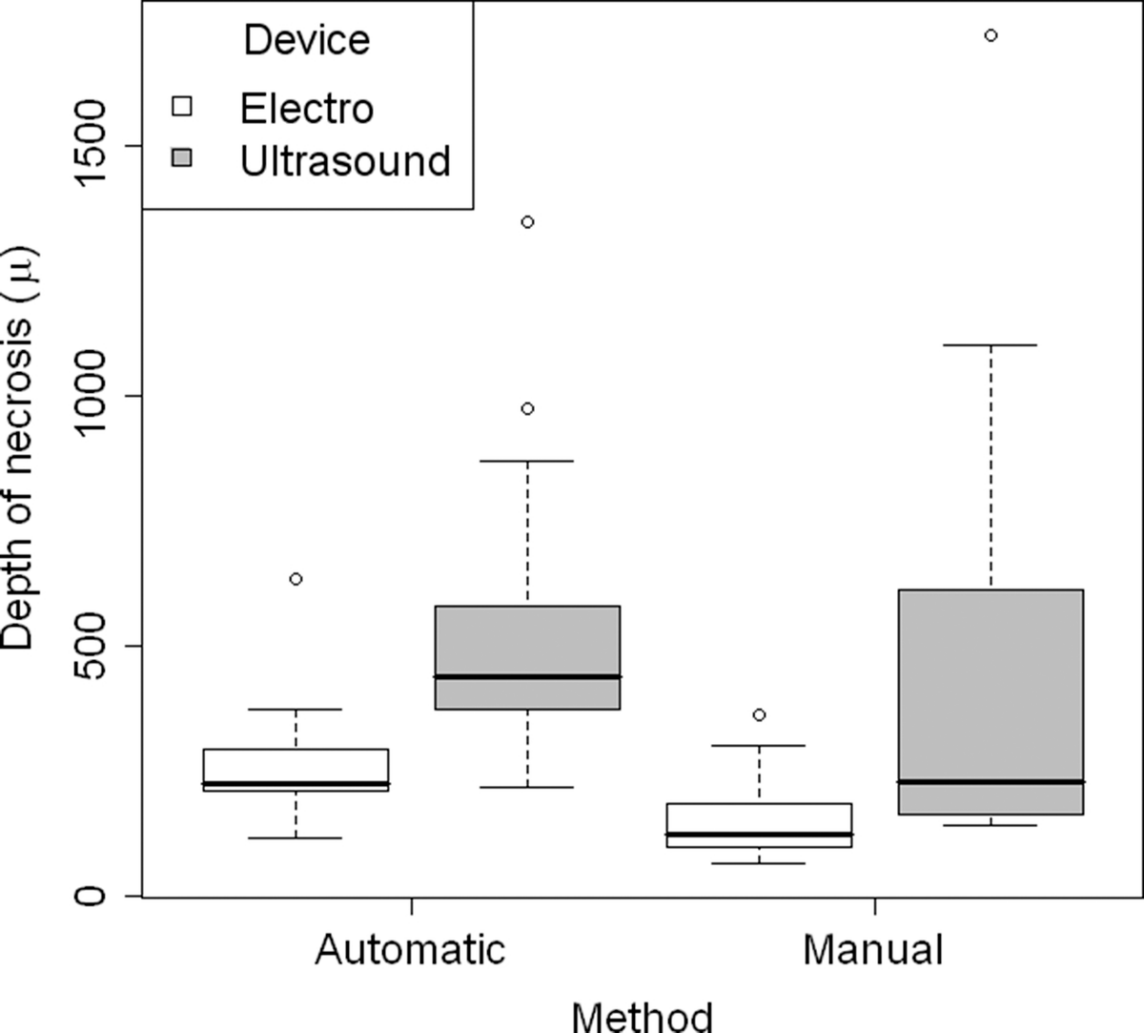
**Box-plot for correlation between depht of necrosis, method and device**. Distribution of the depth of necrosis according to method and device.

Morphometric analyses could only be performed in 70 tissue samples with an exact horizontal resection plane at the cutaneous-subcutaneous junction (Figure 4). In the remaining 30 samples not suitable for analysis, excision was performed to deep in the fatty tissue. Table 1 displays the median depth of coagulation necrosis for all tissue samples. The mean depths (± standard deviation) of coagulation necrosis stratified by mode of excision and dissection tool are illustrated in Figure 5. Depth of necrosis was significantly greater when using UC in comparison to the ME (*p *< 0.01). Though depth of necrosis was also greater when using the automatic compared to the manual mode, this effect, however, was not significant (*p *= 0.85). Furthermore, there was no significant interaction between the mode of excision and the dissection tool (*p *= 0.93). That means the significant tool effect can be regarded to be of the same size under both modes.

**Table 1:** Median depth of coagulation necrosis in single tissue samples

	Ultrasonic scalpel				Monopolar electrocautery			
	***automatic***		***manual***		***automatic***		***manual***	

**Animal**	**A**	**B**	**A**	**B**	**A**	**B**	**A**	**B**

1	416.1	543	1100	n.a.	220	374.5	n.a.	217.8

2	509.7	1346	1721.8	n.a.	196.7	331.2	302.2	185.6

3	868.7	n.a.	612.9	n.a.	240.4	324.1	n.a.	364.1

4	408.4	219.5	645.6	230.5	252.2	224.7	103.8	92.9

5	975.9	n.a.	n.a.	n.a.	633	153.6	116.5	n.a.

6	581.8	375.2	n.a.	177.3	213.1	117.4	72.4	89.8

7	438.4	n.a.	n.a.	n.a.	293.7	222.6	127.5	178.6

8	n.a.	n.a.	n.a.	n.a.	213.4	n.a.	101.7	190.1

9	268.2	244.2	n.a.	n.a.	116.1	253.1	112.6	100.7

10			n.a.	225.6			n.a.	n.a.

11			n.a.	143.3			78	244.2

12			283.8	151			95.3	140.1

13			n.a.	n.a.			250.5	135.4

14			n.a.	141.1			65.9	129.6

15			213.2	163			101.7	153.6

16			n.a.	496.5			n.a.	124

Median Depth of coagulation necrosis in single tissue samples (A and B) measured in micrometer; n.a. = not adequate for morphometric analysis based on inappropriate resection plane

## Discussion

This study shows that both, UC and ME lead to a similar histopathologic pattern of coagulation necrosis at the resection plane. However, the chosen dissection tool significantly affects the depth of this coagulation necrosis with UC generating a greater necrotic margin than ME when used for soft tissue dissection using standard power level settings.

Few previous studies exist specifically on morphological changes of soft tissue caused by UC and ME. Addressing skin and subcutaneous soft tissue dissection in a pig model, Hambely et al. [12] reported significantly less extensive and more localized tissue damages with UC compared to ME. Focusing on quality in contrast to extent of tissue damage, Foschi et al. [13] identified coagulation necrosis to be the predominant thermal injury by scanning and transmission electron microscopy which is consistent with our results.

Examining the efficacy of UC for hemostasis, Diamantis et al. [14] have investigated the safety and efficacy of multiple dissection tools including UC and ME for dissection and coagulation of short gastric vessels in a New Zealand rabbit model. In contrast to our results, they reported a deeper tissue damage caused by ME compared to UC. However, they applied a different, more descriptive approach referring to histological layers but did provide neither exact measurement data nor statistical comparisons. By analyzing the efficacy of UC for the hemostasis of small-, medium- and large-sized arteries in pigs, Harold et al. [15] observed an increase in thermal injury concomitant to increased vessel size. This direct correlation between power level settings, activation time and thermal injury has been reported in more detail by Emam et al. shortly after [11].

These mentioned animal studies share a relevant limitation which is the missing description of morphometric measurement. Our data clearly indicate that both UC and ME do not cause a uniform necrotic zone at the resection margin (Figure 2), most likely not only because of dissection related but local factors like tissue quality and vessel density. This suggestion is supported by the findings of Hoenig et al. [16] They examined the thermal injury of laparosonic coagulating shears with either sharp or blunt tip compared to bipolar electrocautery in a porcine model and observed different extent of injury depending on the type of tissue dissected.

The special contribution of this animal study is that we tried to design an experimental setup that reduces handling related bias as much as possible. In particular, we implemented a randomization-process for sample retrieval, the comparative application of the automatic device versus manual dissection, the excision of 2 samples of each kind (A and B) per animal and multiple measurement points per sample for quantifying the depth of coagulation necrosis.

Facing our result of wider necrotic margin in UC, one might hypothesize that in terms of clinical relevance this might lead to more competent ligation of both, blood vessels and lymphatics. This is supported by Morino et al. [17] who investigated the safety and efficacy of UC compared to ME in laparoscopic colorectal surgery within a prospective randomized clinical trial. They found a significantly lower median intraoperative blood loss for UC. Schmidbauer et al. [18] also reported this convincing coagulating effect with minimal blood loss for UC for the field of liver resection. In contrast, clinical studies evaluating the postoperative seroma rate following breast cancer surgery could not confirm a significant benefit of UC on seroma formation [19,20]. On the other hand, the greater depth of necrosis could also contribute to postoperative nerval dysfunctions when UC is used close to susceptible structures in colorectal surgery. Furthermore, based on our described experimental setup we were able neither to investigate additional relevant but later occurring aspects of tissue alteration in particular inflammatory responses or induction of fibrosis nor to examine the healing sites for differences in wound healing processes or nerval dysfunctions. Therefore, future clinical trials are needed to investigate the clinical relevance of our findings and reason practical recommendations. However, our preliminary data argue for cautious use of UC when susceptible structures are close.

## Conclusions

Our study confirmed that both, UC and ME lead to coagulation necrosis at the resection plane. Operating at standard power levels the depth of this coagulation necrosis is significantly greater when UC is used for soft tissue dissection compared to ME.

## Competing interests

The authors declare that they have no competing interests.

## Authors' contributions

CL and JM conceived of the study and determined the design. JM performed experiments and acquisition of data. BK reviewed all histopathological specimens and performed morphometric measurements. KJ provided statistical analysis. KH and JM interpreted the data and drafted the manuscript. All authors participated in the revision of the manuscript. All authors read and gave final approval of the version submitted for publication.

## Pre-publication history

The pre-publication history for this paper can be accessed here:

http://www.biomedcentral.com/1471-2482/12/3/prepub
